# Reduced Income and Its Associations with Physical Inactivity, Unhealthy Habits, and Cardiac Complications in the Hypertensive Population

**DOI:** 10.3390/ejihpe14080153

**Published:** 2024-08-07

**Authors:** Lucía Carrasco-Marcelo, Damián Pereira-Payo, María Mendoza-Muñoz, Raquel Pastor-Cisneros

**Affiliations:** 1 Department of Financial Economics and Accounting, Faculty of Business, Finance and Tourism, University of Extremadura, Avda. de la Universidad, s/n, 10071 Cáceres, Spain; lucarrascom@unex.es; 2 Health, Economy, Motricity and Education (HEME) Research Group, Faculty of Sport Sciences, University of Extremadura, 10003 Cáceres, Spain; 3 Physical and Health Literacy and Health-Related Quality of Life (PHYQoL), Faculty of Sport Science, University of Extremadura, 10003 Cáceres, Spain; mamendozam@unex.es; 4 Promoting a Healthy Society Research Group (PHeSO), Faculty of Sport Sciences, University of Extremadura, 10003 Cáceres, Spain; raquelpc@unex.es

**Keywords:** alcohol consumption, eating, hypertension, income, self-perceived health

## Abstract

(1) Background: A low socioeconomic status significantly increases the risk of hypertension and its associated cardiovascular diseases due to limited access to healthcare and may be even more accentuated by the presence of unhealthy lifestyle habits. The aim of the present research was to study if associations exist between having a family income under the poverty threshold and having an unhealthy diet, being physically inactive, being an alcohol drinker, perceiving one’s own health as bad, and suffering from congestive heart failure, coronary heart disease, angina pectoris, heart attack, or stroke. Additionally, the odds ratios of having these unhealthy habits and of suffering from the abovementioned cardiac complications of participants under the poverty threshold were calculated. (2) Methods: This cross-sectional study was based on the National Health and Nutrition Examination Survey (NHANES) 2011–2020. The sample comprised 6120 adults with hypertension (3188 males and 2932 females). A descriptive analysis and non-parametric chi-squared tests were used to study the associations. A binary logistic regression model and backward LR method were used to calculate the odds ratios, normalized by age and sex. (3) Results: The chi-squared test showed associations between having a family income under the poverty threshold and being physically inactive (*p* < 0.001), having an unhealthy diet (*p* < 0.001), being an alcohol drinker (*p* < 0.001), perceiving one’s own health as bad (*p* < 0.001), and suffering from congestive heart failure (*p* = 0.002), heart attack (*p* = 0.001), or stroke (*p* = 0.02). A significantly increased odds ratio for these unhealthy habits and cardiac complications, and also for having coronary heart disease and angina pectoris, were found for hypertension sufferers under the poverty threshold. (4) Conclusions: It was confirmed that having a family income under the poverty threshold is associated with perceiving one’s own health as bad, having a series of negative habits in terms of physical activity, diet, and alcohol consumption, and with suffering from congestive heart failure, heart attack, or stroke. Increased odds ratios for these unhealthy habits and these conditions, plus coronary heart disease and angina pectoris, were found for hypertension sufferers under the poverty threshold.

## 1. Introduction

Hypertension, a cardiovascular disease that affects the pressure in the arteries, is considered the highest preventable cause of mortality [1,2]. In low- and middle-income countries (LMICs), it caused 10.4 million deaths per year between 1990 and 2020 [3]. Poverty is considered one of the leading causes of death and disease due to individuals’ limited access to healthcare, equity, security, and lifestyle, among others [4].

Socioeconomic level or status can be defined as a person’s social place within a social group, based on factors such as income, education, or occupation [5]. Recent research has identified socioeconomic disadvantage as an emerging risk factor affecting hypertension [6,7], in addition to demographic characteristics such as sex and age [8].

The literature shows that socioeconomic factors, such as income, wealth, educational level or employment status are related to high blood pressure [9,10,11]. Some studies have found that people with higher income levels have lower rates of cardiovascular disease-related pathologies than people with lower incomes [12].

In this sense, people with high poverty rates who lack economic resources and adequate healthcare are exposed to socio-economic and psychosocial factors such as stress, discrimination or racism [11]. All of the above affect the control of hypertension, a critical chronic disease that can lead to other cardiac pathologies, increasing the risk of individuals who experience both conditions [13,14].

On the other hand, lifestyle habits, defined as behaviors that are always or almost always carried out on a daily basis in stable contexts [15], profoundly affect health and quality of life in both the short and long term [16]. Lifestyle is largely determined by the environmental and socio-economic context in which the person develops, mainly during adolescence or youth [17]. In relation to this, poverty is seen as a key factor in consolidating health patterns that will be maintained over time [18].

Modifiable factors, which refer to lifestyle habits that have the most negative impact on health, are, as follows: physical inactivity [19], an unhealthy diet [20] and high alcohol consumption [21]. People living in poverty have been found to have a higher prevalence of certain unhealthy lifestyles, such as being more physically inactive [22], having difficulty accessing healthy diets [23] and having a higher rate of heavy alcohol consumption [24], leading to a negative impact on the health of this population [25].

Excess hypertension morbidity is more pronounced in people living in poverty compared to their counterparts in a more advantaged socio-economic context [26]. In addition, poorer self-perceived health has been observed in the hypertensive population [27]; thus it is necessary to study the influence of lifestyle on the health of people suffering from this pathology in conditions of poverty.

According to the abovementioned factors, evidence suggests that hypertension sufferers with low incomes may have a higher incidence of certain pathologies and are more prone to including certain unhealthy habits in their lifestyle. Thus, it was hypothesized that having a family income below the poverty threshold is associated with having unhealthy habits regarding physical inactivity, diet, alcohol consumption, and, also, with suffering from certain cardiovascular pathologies. The aim of the present research was to study if associations exist between having a family income under the poverty threshold and having a series of unhealthy habits, perceiving one’s own health as bad, and having congestive heart failure, coronary heart disease, angina pectoris, heart attack, or stroke. The odds ratios of having these unhealthy habits and of suffering from the abovementioned pathologies of those under the poverty threshold were calculated.

## 2. Material and Methods

### 2.1. Design

This is a cross-sectional study based on data from the National Health and Nutrition Examination Survey (NHANES) editions from 2011 to 2020. The National Center for Health Statistics (NCHS) conducts this program, where dietary and health professionals conduct interviews and physical examinations of the selected participants to assess the health and nutritional status of adults and children in the United States [28].

### 2.2. Participants

NHANES participants are selected through a stratified, clustered, four-stage sampling system. Full details in terms of the sample design, calibration and data weighting can be found in [29,30].

The following inclusion criteria were established for participants to be included in this research: having been diagnosed with hypertension, being 18 years old or older, and having valid information for all the variables of interest (family income, physical activity, perception of the own health, diet, alcohol consume, congestive heart failure, coronary heart disease, angina pectoris, heart attack and stroke).

**Figure 1:**
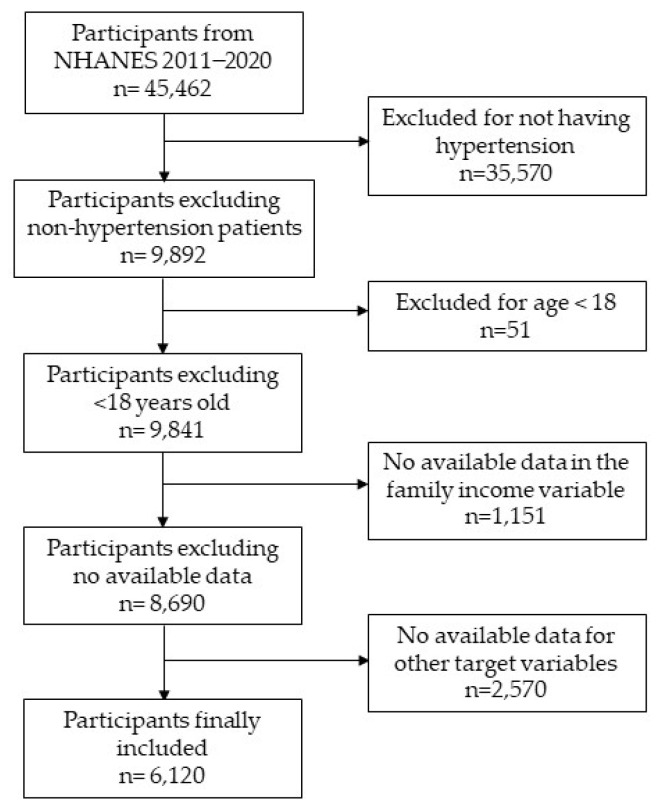
Flow diagram of the participant selection process.

The NHANES 2011–2020 has a total of 45,462 participants (22,472 males and 22,990 females). To make up the sample for the present study, 35,570 participants were excluded because they did not have hypertension; 51 participants were excluded because they were younger than 18 years; and 1151 participants were excluded because they did not have data available on the grouping variable of “family income under the poverty threshold”. In addition, all those participants who did not have valid data on the following target variables were excluded: physical inactivity (536 participants were excluded); negative perception of their own health (9 participants excluded); unhealthy diet (3 participants excluded); 4/5 alcohol drinks a day (1898 participants excluded); congestive heart failure (55 participants excluded); coronary heart disease (32 participants excluded); angina pectoris (19 participants excluded); heart attack (8 participants excluded); and stroke (10 participants excluded) (Figure 1).

Thus, the final sample consisted of 6120 participants (3188 males and 2932 females).

### 2.3. Variables

Age: expressed in years at the moment of interview (item RIDAGEYR in NHANES).

Gender: the possible answers were: “male” or “female” (item RIAGENDR in NHANES).

Race: participants were asked how they self-identified. Possible responses were, as follows: (1) Mexican-American; (2) Other Hispanic; (3) Non-Hispanic White; (4) Non-Hispanic Black; (5) Non-Hispanic Asian; and (5) Other Race and Multiracial. Family income under the poverty threshold: This variable was based on the NHANES ratio of family income to poverty (item INDFMPIR). This ratio ranges from “0” (no income) to “5” (≥5 times the federal poverty level). The poverty threshold is set at “1”, which is the federal poverty level. Thus, participants with a ratio of family income to poverty under “1”, were classified as “family income under the poverty threshold = yes” and those with a number of 1 or above were considered as “family income under the poverty threshold = no”.

Physical inactivity: This variable was derived from the answers of the participants to the Generalized Physical Activity Questionnaire (items PAQ610, PAQ625, PAQ640, PAQ655 and PAQ670 in NHANES) [31]. Participants who reported not performing any days a week of moderate or intense physical activity, and neither walking nor cycling as a way of transportation, were defined as “physically inactive”. Participants who reported performing at least one day a week of these forms of physical activity were defined as “physically active”.

Health perceived as negative (item HUQ010 in NHANES): Participants were asked: “Would you say your health in general is…?”, the possible answers were: “Excellent”, “Very Good”, “Good”, “Fair” or “Poor”. Participants who answered “Excellent”, “Very Good” or “Good”, were defined as not having a negative health perception. Participants who defined their health as “Fair” or “Poor”, were classified as having a negative perception of their health (Health perceived as negative = “yes”).

Unhealthy Diet (item DBQ700 in NHANES): Participants were asked: “In general, how healthy is your overall diet?”. The possible answers were: “Excellent”, “Very Good”, “Good”, “Fair” or “Poor”. Thus, participants who answered “Excellent”, “Very Good” or “Good”, were defined as to not having an unhealthy diet, and those who answered “Fair” or “Poor”, were defined as having an unhealthy diet.

Being an alcohol drinker or former drinker (item ALQ151 in NHANES): Participants were asked: “Was there ever a time or times in your life when you drank four or more drinks of any kind of alcoholic beverage almost every day?”. The possible answers were: “Yes” or “No”.

Congestive Heart Failure (item MCQ160B in NHANES): Participants were asked: “Has a doctor or other health professional ever told you that you had congestive heart failure?”. The possible answers were: “Yes” or “No”.

Coronary Heart disease (item MCQ160C in NHANES): Participants were asked: “Has a doctor or other health professional ever told you that you had coronary heart disease?”. The possible answers were: “Yes” or “No”.

Angina Pectoris (item MCQ160D in NHANES): Participants were asked: “Has a doctor or other health professional ever told you that you had angina pectoris?”. The possible answers were: “Yes” or “No”.

Heart Attack: Participants were asked: “Has a doctor or other health professional ever told you that you had a heart attack (also called myocardial infarction)?”. The possible answers were: “Yes” or “No”, which corresponded to item MCQ160E of the NHANES.

Stroke (item MCQ160F in NHANES.: Participants were asked: “Has a doctor or other health professional ever told you that you had a stroke?”. The possible answers were: “Yes” or “No”.

### 2.4. Statistical Analysis

The Kolmogorov–Smirnov test (*p* < 0.001) and histogram representation were used to test if data followed a normal distribution. Sufficient evidence was not found to assume that data followed a normal distribution. Therefore, non-parametric tests were used in all statistical procedures.

Age, which was the only continuous variable in the study, was presented with median and interquartile ranges (and mean and standard deviation as complementary data). Ordinal variables were presented in their absolute and relative frequencies.

Associations between having or not having an unhealthy habit or a cardiac complication and having or not having a family income below the poverty threshold were studied through the Chi-squared test. The post hoc pairwise z-test for independent proportions was used to analyze the differences in proportions between genders (in the descriptive analysis) and to analyze differences in the proportions of non-healthy habits and cardiac diseases among hypertension sufferers with an income below the poverty threshold or those having a higher income. To study the effect size, Cramer’s V was calculated.

Odds ratios normalized by age and sex were calculated using a binary logistic regression model with the backward LR method. Every binary regression model can be checked in detail in the Supplementary Materials (Tables S1–S9).

All statistical procedures were performed with SPSS software, using the 26th version (IBM SPSS, Chicago, IL, USA). A 0.05 level of significance was assumed.

## 3. Results

Table 1 shows the characterization of the population participating in the NHANES 2011–2020 hypertension sufferers aged 18 years old or above. The median age of the population of study was 61.00 (IDR = 60), with no significant differences among males and females. As for the ordinal variables, the Chi-squared test identified associations between gender and having a family income under the poverty threshold (*p* = 0.002); being physically inactive (*p* < 0.001); perceiving their health as negative (*p* = 0.006); having an unhealthy diet (*p* < 0.001); being a drinker or having been a drinker (*p* < 0.001); and suffering from, or having suffered from, coronary heart disease (*p* < 0.001), angina pectoris (*p* = 0.001) and heart attack (0.007). However, non-associations were found between gender and suffering from congestive heart failure (*p* = 0.104) or stroke (*p* = 0.805).

Significant associations were found in Table 2 between having a family income below the poverty threshold and having a negative perception of one’s own health (*p* < 0.001), and also with being physically inactive (*p* < 0.001), having a bad diet (*p* < 0.001) and being a drinker or having been a drinker (*p* < 0.001). Additionally, significant differences in the proportions between participants with a family income over and under the poverty threshold were observed in the z-test. Thus, an increased prevalence of physical inactivity (38.9% compared to 32.2%), unhealthy diet (43.3% compared to 30.2%), daily drinking of various alcohol beverages (28.5% compared to 18.9%) and having a bad perception of one’s own health (51.7% compared to 29.7%), were found for participants with a family income under the poverty threshold.

In Table 3, regarding the prevalence of cardiac diseases, significant relationships are shown between having a family income below the poverty threshold and the incidence of congestive heart failure (*p* < 0.001), heart attack (*p* < 0.001) and stroke (*p* = 0.04 *). On the other hand, the Chi-squared test did not find associations with the incidence of coronary heart disease (*p* = 0.527) or angina pectoris (*p* = 0.085). An increased prevalence of congestive heart failure (9.4% compared to 6.8%), heart attack (11.1% compared to 8.0%) and stroke (10.5% compared to 7.8%) were found for people with hypertension under the poverty threshold.

**Figure 2:**
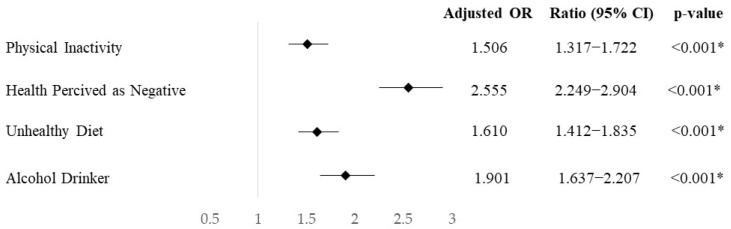
Odds ratio, standardized by age and sex, of having unhealthy habits in the hypertensive population with a family income below the poverty threshold compared to those with a family income equal or above it. * = *p*-value under significance level.

In Figure 2, it can be seen that participants with a family income considered as being under the poverty threshold were found to have an increased odds ratio of perceiving their health as negative (OR= 2.555; CI95% = 2.249–2.904; *p* < 0.001). Additionally, these participants were shown to have greater odds of being physically inactive (OR: 1.506; CI95%: 1.317–1.722; *p* < 0.001), having a non-healthy diet (OR: 1.610; CI95%: 1.412–1.835; *p* < 0.001), and being a drinker or former drinker (OR: 1.901; CI95%: 1.637–2.207; *p* < 0.001).

**Figure 3:**
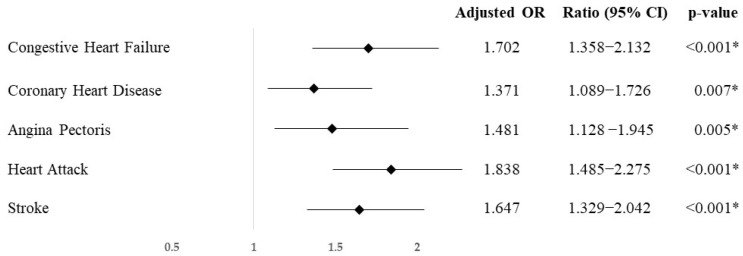
Odds ratio, standardized by age and sex, of having cardiac pathologies of the hypertensive population with a family income below the poverty threshold compared to those with an income equal or above it. * = *p*-value under de level of significance.

At the same time, in Figure 3, the odds ratio normalized by age and sex shows that participants with a family income under the poverty threshold have increased odds of suffering from congestive heart failure (OR = 1.702; CI95% = 1.358–2.132; *p* < 0.001), coronary heart disease (OR = 1.371; CI95% = 1.089–1.726, *p* = 0.007), angina pectoris (OR = 1.481; CI95% = 1.128–1.945; *p* = 0.005), heart attack (OR = 1.838; CI95% = 1.485–2.275; *p* < 0.001), and stroke (OR = 1.647; CI95% = 1.329–2.042; *p* < 0.001).

In Table 4, a binary logistic regression model for suffering from any of the studied cardiac pathologies (congestive heart failure, coronary heart disease, angina pectoris, heart attack and stroke) is shown. Among the independent variables included in the model, age (*p* < 0.001), race (*p* < 0.001), gender (*p* < 0.001), physical activity (*p* < 0.001), health perception (*p* < 0.001), family income (*p* = 0.001) and being an alcohol drinker or former drinker (*p* < 0.001) were found to be significant. On the other hand, having an unhealthy diet (*p* = 0.132) was the only independent variable not significant in the model.

## 4. Discussion

The objective of this work was to study if associations exist between having a family income under the poverty threshold and engaging in unhealthy behavior, having a negative perception of one’s own health, and suffering from, or having suffered from, a series of cardiac pathologies. The results of the study show that there are associations between being under the poverty threshold with being physically inactive, describing one’s diet as unhealthy, being or have been an alcohol drinker, perceiving one’s own health as negative, and suffering from congestive heart failure, or having a heart attack or stroke. Additionally, the odds of suffering from the cited cardiac pathologies, plus angina pectoris and strokes, incurred from the abovementioned unhealthy habits, were increased for participants under the poverty threshold compared to those with a higher family income.

Regarding the perception of one’s own health, it was found that being above or below the poverty line is associated with having a positive or negative perception of health. In addition, we found that people who are below the poverty line have a higher chance of perceiving their health as negative, according to the odds ratio analysis. In this sense, other studies have found associations between health perception and socioeconomic status, showing that variations in self-perceived health as a function of socioeconomic variables may be pathways that explain socioeconomic gradients in mortality and morbidity [32]. It is important to consider the variable of self-perceived health, as it is directly related to physical, emotional, and cognitive components [33], as expressed in the present study.

In terms of lifestyle habits, this study identified a series of unhealthy behaviors associated with being below the poverty threshold, such as being physically inactive, having an unhealthy diet and drinking or having four or more alcohol drinks on a daily basis. The odds of being inactive, a drinker, and having an unhealthy diet were increased for those under the poverty threshold. With regard to physical activity, people with very low incomes or living in poverty were found to have a higher prevalence of physical inactivity [34], probably because they have less access to places that encourage physical activity, such as green spaces, cycle paths, or safe neighborhoods [35]. In this context, low-income earners tend to live in neighborhoods with higher crime rates than higher-income earners [36,37]. Given that there are higher odds ratios for being physically active in low-crime areas, it has been shown that having a higher salary is associated with being more physically active [38], which is in line with our findings.

Regarding diet, some studies showed that socioeconomic status influences the prevalence of poor-quality or unhealthy diets [39]. In turn, diet and nutrition are very important factors influencing the prevention and detection of non-communicable diseases, such as cardiovascular disease or type II diabetes [40,41], making it necessary to change to healthier diets in order to reduce medical costs, prevent these types of diseases, and, ultimately, improve the quality of life. Therefore, in relation to the results of our study, it has been shown that people living in poverty or with a very low income have higher odds of having an unhealthy diet [42], probably due to a lack of knowledge about nutritional habits, the more affordable prices of fast food, or exposure to neighborhood establishments selling this type of food [43,44]. Additionally, it has also been shown that higher levels of education are positively associated with better dietary habits [45] and that socio-economically disadvantaged areas are more likely to follow less healthy dietary patterns, mainly due to the population’s access to food [46,47], emphasizing the fact that dietary behaviors remain a social gradient today.

Focusing on alcohol consumption, liver disease associated with heavy alcohol consumption is one of the fastest growing causes of mortality and morbidity worldwide [48]. Several studies have shown the increased prevalence of being an alcoholic for those with an unfavorable socioeconomic status; this is especially increasing among women [49,50]. Previous studies have found a higher odds ratio of high alcohol consumption for those with a very low income or who live in poverty [51,52], which is consistent with the findings of the present study.

When it comes to cardiac pathologies, the results show that having a family income below the poverty threshold is associated with an increased prevalence of congestive heart failure, heart attack, and stroke. However, it is not associated with suffering from coronary heart disease or angina pectoris. The analysis of the odds ratio normalized by age and sex showed that participants in difficult economic situations have a higher risk of suffering from congestive heart failure, coronary heart disease, angina pectoris, heart attack, and stroke. Associations have been observed between socioeconomic status and the incidence of heart diseases [53], in which socioeconomic determinants may have an impact on the pathophysiology of the disease. At the same time, people with an unfavorable economic situation have been found to be at a higher risk of heart disease due to several factors discussed in previous studies. Access to healthcare remains one of the most important determinants, as prolonged lack of access to preventive care and screening for cardiovascular disease determines the prevalence of the disease in economically disadvantaged populations [54]. Confirming our results, the incidence of heart failure [55], heart attack [56], and stroke [57] was shown to be associated with socioeconomic status, with an increased prevalence of cardiac conditions for those in unfavorable economic situations. On the other hand, and in contrast to our results, premature incidences of coronary heart disease were found to be associated with a low socioeconomic status [58].

On the other hand, another axis to consider is the chronic physiological stress experienced by people with socio-economic difficulties. This situation leads to activation of the sympathetic nervous system and the hypothalamic–pituitary axis [59]. These are associated with increased markers of systemic inflammation and abnormalities in glucose and lipid metabolism [60], which are predictors of cardiovascular disease.

The geographic area of residence of the most socioeconomically disadvantaged populations is also associated with higher rates of cardiovascular disease prevalence, hospitalization, and death [61], compared to areas with higher socioeconomic levels.

One limitation of the present investigation is that lifestyle habits were not quantified objectively; rather, it was the participants themselves who stated whether they were physically active (through the IPAQ questionnaire), and who reported on their alcohol consumption and the quality of their diet.

Since this is a cross-sectional study, due to the nature of this type of research, we cannot establish cause–effect relationships between the variables studied. For this reason, the objective of this work was to study the associations between the variables.

## 5. Conclusions

Considering the significant associations found in the present study, the results may serve as a reference for public education and health institutions to focus education and training programs on healthy behaviors and physical activity interventions in economically disadvantaged populations.

This study aims to reduce the risk of heart disease by modifying the unhealthy habits, such as by increasing physical activity, improving diet and reducing the alcohol consumption, of very low-income families. At the same time, improving these behaviors would allow them to perceive their health in a positive way.

## Figures and Tables

**Table 1:** Characterization of the NHANES 2011-2020 hypertension sufferers aged 18 years old or above.

Variable	General Population (*n* = 6120)		Males (*n =* 3188)		Females (*n =* 2932)		*p* *
Age							
Mean (SD)	58.98 (14.83)		58.74 (15.02)		59.25 (14.62)		-
Median (IQR)	61.00 (60)		61.00 (60)		61.00 (60)		0.325
Family income under the poverty threshold	General Population (*n* = 6120)		Males (*n =* 3188)		Females (*n =* 2932)		*p*
No	4869	79.6%	2586	81.1%	2283 *	77.9%	0.002 *
Yes	1251	20.4%	602	18.9%	649 *	22.1%	
Physically inactive	General Population (*n* = 6120)		Males (*n =* 3188)		Females (*n =* 2932)		*p*
No	4869	79.6%	2586	81.1%	2283 *	77.9%	<0.001 *
Yes	1251	20.4%	602	18.9%	649 *	22.1%	
Health perceived as negative	General Population (*n* = 6120)		Males (*n =* 3188)		Females (*n =* 2932)		*p*
No	2091	34.2%	1038	32.6%	1053 *	35.9%	0.006
Yes	4029	65.8%	2150	67.4%	1879 *	64.1%	
Unhealthy Diet	General Population (*n* = 6120)		Males (*n =* 3188)		Females (*n =* 2932)		*p*
No	2011	32.9%	963	30.2%	1048 *	35.7%	<0.001 *
Yes	4109	67.1%	2225	69.8%	1884 *	64.3%	
Being an alcohol drinker or having been a drinker	General Population (*n* = 6120)		Males (*n =* 3188)		Females (*n =* 2932)		*p*
No	4843	79.1%	2221	69.7%	2622 *	89.4%	<0.001 *
Yes	1277	20.9%	967	30.3%	310 *	10.6%	
Congestive Heart Failure	General Population (*n* = 6120)		Males (*n =* 3188)		Females (*n =* 2932)		*p*
No	5670	92.6%	2937	92.1%	2733	93.2%	0.104
Yes	450	7.4%	251	7.9%	199	6.8%	
Coronary Heart disease	General Population (*n* = 6120)		Males (*n =* 3188)		Females (*n =* 2932)		*p*
No	5601	91.5%	2838	89.0%	2763 *	94.2%	<0.001 *
Yes	519	8.5%	350	11.0%	169 *	5.8%	
Angina Pectoris	General Population (*n* = 6120)		Males (*n =* 3188)		Females (*n =* 2932)		*p*
No	5809	94.9%	3003	94.2%	2806 *	95.7%	0.007
Yes	311	5.1%	185	5.8%	126 *	4.3%	
Heart Attack	General Population (*n* = 6120)		Males (*n =* 3188)		Females (*n =* 2932)		*p*
No	5590	91.3%	2835	88.9%	2755 *	94.0%	0.001 *
Yes	530	8.7%	353	11.1%	177 *	6.0%	
Stroke	General Population (*n* = 6120)		Males (*n =* 3188)		Females (*n =* 2932)		*p*
No	5610	91.7%	2925	91.8%	2685	91.6%	0.805
Yes	510	8.3%	263	8.2%	247	8.4%	

SD = standard deviation; IQR = interquartile range; *p* = *p*-value of the Chi-square test; *p* * = *p*-value of the Mann–Whitney U test.

**Table 2:** Associations between various unhealthy behaviors and having an income above or below the poverty line.

	Equal/Above Poverty Threshold		Under Poverty Threshold		X2	Phi	*p*-Value
Physical Inactivity	N	(%)	N	(%)			
No	3300	67.8%	764 *	61.1%	20.06	−0.057	<0.001
Yes	1569	32.2%	487 *	38.9%			
Health perceived as negative							
No	3425	70,3%	604 *	48.3%	215.36	−0.188	<0.001
Yes	1444	29,7%	647 *	51.7%			
Unhealthy diet							
Yes	3400	69,8%	709 *	56.7%	78.07	−0.113	<0.001
No	1469	30,2%	542 *	43.3%			
Being an alcohol drinker or former drinker							
No	3948	81.1%	895 *	71.5%	54.88	−0.095	<0.001
Yes	921	18.9%	356 *	28.5%			

“Equal/Above poverty threshold” = participants with a family income above or equal to the poverty threshold; “Under poverty threshold” = participants with a family income below the poverty threshold; * = *p*-value for the Chi-squared test under level of significance.

**Table 3:** Associations of suffering from, or having suffered from, congestive heart failure, coronary heart disease, angina pectoris, heart attack and stroke with having an income below the poverty line.

	Equal/Above Poverty Threshold		Under Poverty Threshold		X2	Phi	*p*-Value
Congestive Heart Failure	N	(%)	N	(%)			
No	4537	93.2%	1133 *	90.6%	9.98	0.040	0.002 *
Yes	332	6.8%	118 *	9.4%			
Coronary heart disease							
No	4460	91.6%	1141	91.2%	0.198	0.006	0.656
Yes	409	8.4%	110	8.8%			
Angina pectoris							
No	4633	95.2%	1176	94.0%	2.720	0.021	0.099
Yes	236	4.8%	75	6.0%			
Heart attack							
No	4478	92.0%	1112 *	88.9%	11.94	0.044	0.001 *
Yes	391	8.0%	139 *	11.1%			
Stroke							
No	4490	92.2%	1120 *	89.5%	9.41	0.039	0.02 *
Yes	379	7.8%	131 *	10.5%			

“Equal/Above poverty threshold” = participants with a family income above or equal to the poverty threshold; “Under poverty threshold” = participants with a family income below the poverty threshold; * = *p*-value for the Chi-squared test under de level of significance.

**Table 4:** Binary logistic regression model with the backward LR method, of suffering from any of the studied cardiac pathologies, including as independent variables, age, gender, physical activity, health perception, family income, being an alcohol drinker or former drinker, and diet quality.

							95% CI	
Suffering Any of the Studied Cardiac Pathologies (Yes)	B	S.E.	Wald	df	Sig.	Exp (B)	Lower	Upper
Age	0.055	0.003	370.939	1	<0.001	1.057	1.051	1.063
Gender (female)	−0.388	0.071	30.085	1	<0.001	0.678	0.590	0.779
Race (Mexican American)			57.330	5	<0.001			
Race (Other Hispanic)	−0.848	0.217	15.324	1	<0.001	0.428	0.280	0.655
Race (Non-Hispanic White)	−0.489	0.211	5.395	1	0.020	0.613	0.406	0.926
Race (Non-Hispanic Black)	−0.014	0.183	0.006	1	0.939	0.986	0.688	1.412
Race (Non-Hispanic Asian)	−0.198	0.187	1.127	1	0.288	0.820	0.569	1.182
Race (Other Race and Multiracial)	−0.774	0.247	9.809	1	0.002	0.461	0.284	0.749
Physically inactive (no)	−0.248	0.071	12.293	1	<0.001	0.780	0.679	0.896
Health perceived as negative (no)	−1.075	0.072	226.051	1	<0.001	0.341	0.297	0.393
Family income under the poverty threshold (no)	−0.320	0.083	14.751	1	<0.001	0.726	0.617	0.855
Being an alcohol drinker or having been a drinker (no)	−0.315	0.081	15.075	1	<0.001	0.730	0.622	0.856
Constant	−3.038	0.258	138.434	1	<0.001	0.048		
Unhealthy Diet †					0.191			

S.E. = Standard error; df = degree of freedom; sig. = level of significance; † = variable out of the equation, because *p* > 0.05.

## Data Availability

Datasets will be made available upon reasonable request.

## Supplementary Materials

The following supporting information can be downloaded at: https://www.mdpi.com/article/10.3390/ejihpe14080153/s1, Table S1: Binary logistic regression model for being physically inactive; Table S2: Binary logistic regression model for having a negative perception of their own health; Table S3: Binary logistic regression model for perceiving their diet as negative; Table S4: Binary logistic regression model for high alcohol consumption; Table S5: Binary logistic regression model for having congestive heart failure; Table S6: Binary logistic regression model for having coronary heart disease; Table S7: Binary logistic regression model for having angina pectoris; Table S8: Binary logistic regression model for having heart attack; Table S9: Binary logistic regression model for having a stroke.
