# Supplementary material for: Reduced Income and Its Associations with Physical Inactivity, Unhealthy Habits, and Cardiac Complications in the Hypertensive Population
Source: Eur J Investig Health Psychol Educ. 2024 Aug 7;14(8):2300–13. doi: 10.3390/ejihpe14080153 (PMC11353786; doi:10.3390/ejihpe14080153)
Supplement: Supplementary file 1 [file ejihpe-14-00153-s001.zip › ejihpe-3099232-supplementary.pdf]

Table S1. Binary logistic regression model for being physically inactive.

							95% C.I.for EXP(B)	
	B	S.E.	Wald	df	Sig.	Exp(B)	Lower	Upper
Gender (Male)	0.534	0.051	111.007	1	<0.001	1.706	1.545	1.884
Age in years at screening	-0.033	0.002	317.666	1	<0.001	0.968	0.964	0.971
PorDebajoRatio(1)	0.400	0.060	44.953	1	<0.001	1.491	1.327	1.676
Constant	2.045	0.121	287.069	1	<0.001	7.732		

S.E.= standard error; df= degree of freedom.

Table S2. Binary logistic regression model for having a negative perception of their own health.

							95% C.I.for EXP(B)	
	B	S.E.	Wald	df	Sig.	Exp(B)	Lower	Upper
Gender (Male)	0.159	0.050	10.199	1	0.001	1.173	1.063	1.293
Age in years at screening	-0.004	0.002	5.643	1	0.018	0.996	0.993	0.999
PorDebajoRatio(1)	0.953	0.057	276.703	1	<0.001	2.594	2.319	2.903
Constant	0.069	0.111	0.382	1	0.537	1.071		

S.E.= standard error; df= degree of freedom.

Table S3. Binary logistic regression model for perceiving their diet as negative.

							95% C.I.for EXP(B)	
	B	S.E.	Wald	df	Sig.	Exp(B)	Lower	Upper
Gender (Male)	0.268	0.051	27.251	1	<0.001	1.307	1.182	1.445
Age in years at screening	0.028	0.002	271.635	1	<0.001	1.029	1.025	1.032
PorDebajoRatio(1)	0.485	0.059	67.923	1	<0.001	1.624	1.447	1.823
Constant	-1.405	0.113	155.383	1	<0.001	0.245		

S.E.= standard error; df= degree of freedom.

Table S4. Binary logistic regression model for high alcohol consumption.

							95% C.I.for EXP(B)	
	B	S.E.	Wald	df	Sig.	Exp(B)	Lower	Upper
Gender (Male)	-1.343	0.073	341.329	1	<0.001	0.261	0.226	0.301
PorDebajoRatio(1)	0.651	0.076	72.566	1	<0.001	1.918	1.651	2.228
Constant	1.659	0.080	427.860	1	<0.001	5.252		

S.E.= standard error; df= degree of freedom.

Table S5. Binary logistic regression model for having congestive heart failure.

							95% C.I.for EXP(B)	
	B	S.E.	Wald	df	Sig.	Exp(B)	Lower	Upper
Gender (Male)	-0.188	0.091	4.293	1	0.038	0.829	0.694	0.990
Age in years at screening	-0.050	0.004	168.745	1	<0.001	0.952	0.944	0.959
PorDebajoRatio(1)	0.544	0.101	28.804	1	<0.001	1.723	1.413	2.102
Constant	5.373	0.266	407.175	1	<0.001	215.423		

S.E.= standard error; df= degree of freedom.

Table S6. Binary logistic regression model for having coronary heart disease.

							95% C.I.for EXP(B)	
	B	S.E.	Wald	df	Sig.	Exp(B)	Lower	Upper
Gender (Male)	-0.785	0.090	76.569	1	<0.001	0.456	0.383	0.544
Age in years at screening	-0.067	0.004	280.819	1	<0.001	0.935	0.928	0.942
PorDebajoRatio(1)	0.293	0.104	7.944	1	0.005	1.341	1.093	1.644
Constant	6.936	0.289	575.352	1	<0.001	1028.333		

S.E.= standard error; df= degree of freedom.

Table S7. Binary logistic regression model for having angina pectoris.

							95% C.I.for EXP(B)	
	B	S.E.	Wald	df	Sig.	Exp(B)	Lower	Upper
Gender (Male)	-0.380	0.111	11.788	1	0.001	0.684	0.551	0.850
Age in years at screening	-0.041	0.004	83.498	1	<0.001	0.960	0.952	0.969
PorDebajoRatio(1)	0.350	0.126	7.686	1	0.006	1.419	1.108	1.818
Constant	5.483	0.312	309.072	1	<0.001	240.657		

S.E.= standard error; df= degree of freedom.

Table S8. Binary logistic regression model for having heart attack.

							95% C.I.for EXP(B)	
	B	S.E.	Wald	df	Sig.	Exp(B)	Lower	Upper
Gender (Male)	-0.845	0.091	87.080	1	<0.001	0.430	0.360	0.513
Age in years at screening	-0.056	0.004	221.525	1	<0.001	0.945	0.938	0.952
PorDebajoRatio(1)	0.526	0.100	27.930	1	<0.001	1.692	1.392	2.056
Constant	6.077	0.268	512.756	1	<0.001	435.620		

S.E.= standard error; df= degree of freedom.

Table S9. Binary logistic regression model for having a stroke.

							95% C.I.for EXP(B)	
	B	S.E.	Wald	df	Sig.	Exp(B)	Lower	Upper
Age in years at screening	-0.046	0.004	173.695	1	<0.001	0.955	0.948	0.961
PorDebajoRatio(1)	0.403	0.097	17.231	1	<0.001	1.497	1.237	1.810
Constant	5.022	0.241	432.971	1	<0.001	151.658		

S.E.= standard error; df= degree of freedom.
